# Trousseau syndrome presenting with recurrent multi-territory infarctions: a case report of two patients highlighting divergent outcomes and dynamic hypercoagulability under direct oral anticoagulants and low-molecular-weight heparin

**DOI:** 10.3389/fcvm.2026.1834397

**Published:** 2026-05-20

**Authors:** Gangyu Ding, Jianhua Xu, Meifen Yao

**Affiliations:** 1 Department of Neurology, Jiading District Central Hospital Affiliated Shanghai University of Medicine & Health Sciences, Shanghai, China; 2 Department of Neurology, Shanghai East Hospital, School of Medicine, Tongji University, Shanghai, China

**Keywords:** case report, D-dimer, direct oral anticoagulants, hypercoagulability, ischemic stroke, low-molecular-weight heparin, multi-territory infarction, Trousseau syndrome

## Abstract

**Background:**

Trousseau syndrome is a cancer-associated hypercoagulable state characterized by recurrent thromboembolic events, including ischemic stroke. Optimal anticoagulation strategies remain controversial, particularly regarding the effectiveness of direct oral anticoagulants (DOACs) compared with low-molecular-weight heparin (LMWH).

**Case presentation:**

We report two patients with Trousseau syndrome who presented with recurrent multi-territory cerebral infarctions despite ongoing DOAC therapy. Case 1 was a 66-year-old man with active intrahepatic cholangiocarcinoma who developed repeated ischemic strokes and deep vein thrombosis while receiving rivaroxaban. After switching to LMWH, D-dimer levels decreased markedly. However, anticoagulation was de-escalated in the context of apparent normalization of D-dimer, followed by recurrent stroke, subsequent discontinuation of anticoagulation, and a rapid surge in D-dimer levels, ultimately resulting in death. Case 2 was a 74-year-old man with suspected recurrent metastatic gastric adenocarcinoma who developed ischemic stroke while on rivaroxaban. Following transition to LMWH, D-dimer levels progressively declined, and the patient remained clinically stable without recurrence during follow-up. A longitudinal timeline analysis integrating biomarker dynamics, treatment transitions, and clinical events demonstrated distinct patterns between the two cases, suggesting a potential discordance between D-dimer levels and underlying hypercoagulable activity.

**Conclusion:**

These cases highlight the dynamic nature of hypercoagulability in Trousseau syndrome. Normalization of D-dimer alone may not reliably indicate sustained control of malignancy-associated hypercoagulability and should not be used in isolation to guide anticoagulation de-escalation. LMWH may provide more consistent control in selected high-risk patients, although causal inference cannot be established from this report.

## Highlights

- Trousseau syndrome may present with recurrent multi-territory infarctions despite DOAC therapy.
- Normalization of D-dimer does not necessarily indicate resolution of hypercoagulability.
- De-escalation or interruption of anticoagulation may lead to rapid thrombotic deterioration.
- Low-molecular-weight heparin may provide more consistent control of hypercoagulability in selected patients.
- Timeline analysis reveals dynamic relationships between biomarkers, treatment, and outcomes.

## Introduction

Trousseau syndrome is a well-recognized paraneoplastic phenomenon characterized by a systemic hypercoagulable state associated with malignancy, particularly adenocarcinomas. It commonly manifests as venous thromboembolism, arterial thrombosis, and recurrent ischemic stroke, often involving multiple vascular territories (1–4). These thrombotic events are thought to be driven by tumor-related mechanisms, including mucin secretion, activation of selectin-mediated pathways, platelet aggregation, and widespread microthrombosis (3, 5, 6).

Ischemic stroke in the context of Trousseau syndrome is clinically distinctive, frequently presenting as multifocal infarctions affecting both anterior and posterior circulations, and is often associated with markedly elevated biomarkers of hypercoagulability, such as D-dimer (7–10). Notably, the severity of thrombotic risk may not correlate with traditional vascular risk factors or initial neurological deficits, complicating early risk stratification and management (10, 11).

Optimal anticoagulation strategies for Trousseau syndrome remain controversial. While direct oral anticoagulants (DOACs) have become increasingly used in cancer-associated thrombosis because of their convenience and acceptable safety profiles, emerging evidence suggests that they may be insufficient to fully control malignancy-associated hypercoagulability in some patients, particularly in the setting of cancer-associated stroke or recurrent thrombosis (12–15). In contrast, low-molecular-weight heparin (LMWH) has been proposed to exert broader antithrombotic effects, potentially through both anticoagulant activity and interference with tumor-mediated pathways, including selectin-dependent interactions (5, 16–18). However, comparative clinical data in Trousseau syndrome remain limited, and real-world evidence regarding dynamic treatment responses is still lacking (12, 15, 19).

Furthermore, the temporal relationship between biomarker fluctuations, therapeutic adjustments, and clinical outcomes in Trousseau syndrome has not been well characterized. Serial and post-treatment D-dimer levels have been associated with recurrent stroke and poor outcomes in cancer-associated stroke, but it remains unclear whether normalization of D-dimer reliably indicates resolution of the hypercoagulable state or can safely guide de-escalation of anticoagulation therapy (15, 20–23).

Here, we report two patients with Trousseau syndrome who developed recurrent multi-territory cerebral infarctions despite ongoing DOAC therapy, yet demonstrated divergent clinical outcomes following different anticoagulation strategies. By integrating serial D-dimer measurements, treatment transitions, and clinical events within a structured longitudinal framework, this report aims to explore the potential mismatch between biomarker dynamics and true hypercoagulable activity, a phenomenon that remains insufficiently characterized.

## Case presentation

### Case 1

#### Patient information

A 66-year-old man with a history of active intrahepatic cholangiocarcinoma diagnosed approximately 6 months earlier was admitted with speech impairment persisting for 4 days. Approximately one month prior to admission, he had experienced multiple cerebral infarctions and had been treated with oral rivaroxaban. During the same period, a left popliteal deep vein thrombosis was identified. He had no history of hypertension, diabetes mellitus, or coronary artery disease.

#### Clinical findings

On admission, his blood pressure was 112/63 mmHg. He was conscious but exhibited impaired comprehension and answered questions irrelevantly. Neurological examination revealed sensory aphasia with relatively preserved articulation. Cranial nerve examination was largely unremarkable. Muscle strength was grade 5 in all extremities, with normal muscle tone and preserved tendon reflexes. The National Institutes of Health Stroke Scale (NIHSS) score was 6, and the modified Rankin Scale (mRS) score was 3.

#### Diagnostic assessment

Laboratory testing demonstrated moderate anemia (hemoglobin 88 g/L) and markedly elevated D-dimer levels (3.44–4.16 mg/L). Tumor markers were significantly elevated, including carcinoembryonic antigen (CEA) at 277.92 ng/mL and carbohydrate antigen 19-9 (CA19-9) >1,200,000 U/mL.

**Figure 1:**
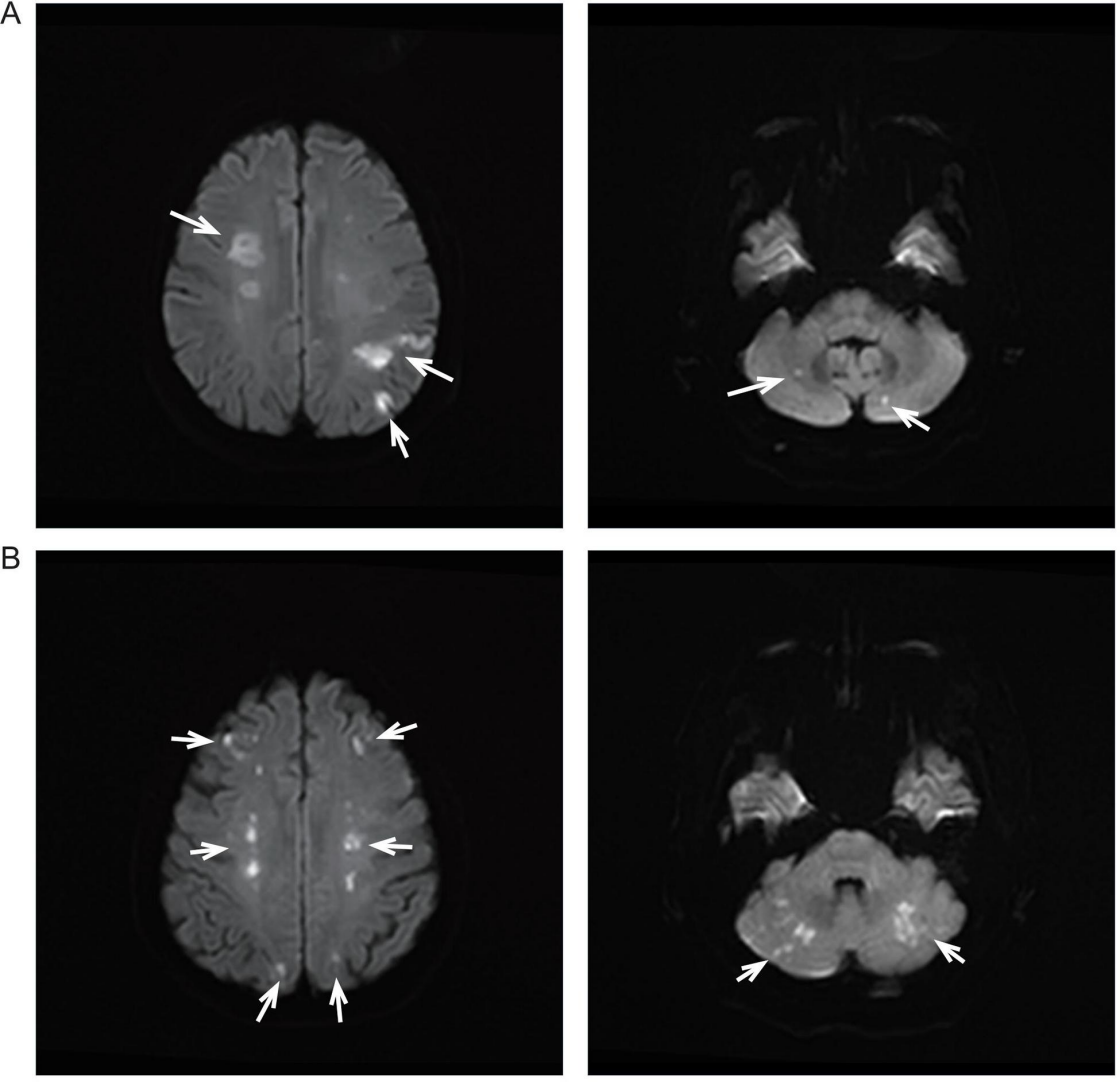
Diffusion-weighted imaging (DWI) findings in two patients with malignancy-associated stroke. **(A)** Case 1: Representative supratentorial (upper) and infratentorial (lower) DWI images demonstrate multiple acute infarcts involving bilateral cortical and subcortical regions as well as the cerebellum, indicating both anterior and posterior circulation involvement. **(B)** Case 2: DWI images reveal numerous scattered acute infarcts in the bilateral cerebral hemispheres and cerebellum, showing a diffuse multi-territory distribution suggestive of embolic infarctions associated with malignancy-related hypercoagulability. White arrows indicate representative lesions. The diffuse multi-territory distribution involving both anterior and posterior circulations is suggestive of embolic showering associated with malignancy-related hypercoagulability.

Brain magnetic resonance imaging (MRI) revealed multiple acute infarctions involving the corona radiata, centrum semiovale, cortical regions of the temporal and parietal lobes, and bilateral cerebellar hemispheres, consistent with a multi-territory infarction pattern involving both anterior and posterior circulations (Figure 1A). Computed tomography angiography (CTA) showed no significant large-vessel stenosis.

Given the presence of active malignancy, recent venous thrombosis, recurrent multi-territory infarctions, and elevated D-dimer levels, a clinical diagnosis of Trousseau syndrome was established.

#### Therapeutic intervention and clinical course

Given the clinical suspicion of Trousseau syndrome and the presence of swallowing impairment, anticoagulation was transitioned from rivaroxaban to low-molecular-weight heparin (LMWH; enoxaparin 40 mg (0.4 mL) subcutaneously every 12 h), which is generally preferred in malignancy-associated hypercoagulability. Following initiation of LMWH, D-dimer levels progressively decreased and normalized, suggesting apparent but potentially transient control of the hypercoagulable state.

After clinical stabilization, anticoagulation was de-escalated in the context of overall clinical improvement, including apparent normalization of D-dimer levels, and rivaroxaban was resumed after discharge. This decision was also influenced in part by patient and family preference to avoid long-term subcutaneous injections.

#### Follow-up and outcomes

Approximately 3 weeks after discharge, the patient developed a third episode of cerebral infarction and was readmitted. At readmission, neurological impairment had significantly worsened (NIHSS score 17). Anticoagulation was discontinued and replaced with antiplatelet therapy (aspirin 100 mg daily) because of concerns regarding hemorrhagic risk.

This was followed by a rapid and marked increase in D-dimer levels (up to 41.96 mg/L), indicating severe exacerbation of hypercoagulability. Despite supportive management, the patient's condition deteriorated rapidly, and he ultimately died of brain herniation.

### Case 2

#### Patient information

A 74-year-old man was admitted with left-sided weakness of more than 1 day in duration. His medical history included hypertension, type 2 diabetes mellitus, and gastrectomy for gastric cancer more than 20 years earlier. Approximately 2 weeks before admission, he had been diagnosed with deep vein thrombosis and was receiving rivaroxaban.

#### Clinical findings

On admission, he was conscious and cooperative. Neurological examination revealed mild left-sided weakness, with muscle strength graded as IV in the left upper limb and V− in the left lower limb. Cranial nerve examination showed left-beating nystagmus and slight tongue deviation to the right. The NIHSS score was 1, and the mRS score was 2.

#### Diagnostic assessment

Laboratory tests demonstrated thrombocytopenia (platelet count 77 × 10^9^/L) and markedly elevated D-dimer levels (24.02 mg/L, later decreasing to 6.87 mg/L). Tumor markers were significantly elevated, including CEA at 798.10 ng/mL and CA19-9 >1,200,000 U/mL.

Brain MRI revealed multiple acute infarctions involving bilateral frontal, parietal, and occipital lobes, cerebellar hemispheres, thalamus, and right temporal lobe, consistent with multi-territory embolic infarctions (Figure 1B). CTA showed no large-vessel occlusion.

Whole-body PET-CT demonstrated hypermetabolic lesions in the gastric remnant, liver, and multiple lymph nodes, suggestive of recurrent metastatic malignancy. Based on these findings, a clinical diagnosis of Trousseau syndrome was made.

#### Therapeutic intervention and clinical course

Despite ongoing rivaroxaban therapy, the occurrence of ischemic stroke prompted a change in anticoagulation strategy. Given the clinical diagnosis of Trousseau syndrome, in which low-molecular-weight heparin is generally preferred, together with the apparent inadequate efficacy of oral anticoagulation as evidenced by stroke occurrence during treatment, anticoagulation was transitioned to low-molecular-weight heparin (enoxaparin 40 mg (0.4 mL) subcutaneously every 12 h). Following this adjustment, D-dimer levels decreased markedly and subsequently normalized, indicating apparent control of the hypercoagulable state.

During hospitalization, biopsy was considered for tumor evaluation but deferred based on multidisciplinary clinical evaluation of high procedural risk in the setting of active hypercoagulability. Anticoagulation with low-molecular-weight heparin was therefore continued.

After discharge, the patient underwent biopsy at an external institution, and histopathological examination confirmed poorly differentiated adenocarcinoma.

#### Follow-up and outcomes

With continued LMWH therapy, the patient remained clinically stable without recurrent thromboembolic events during follow-up, and D-dimer levels remained within the normal range.

**Figure 2:**
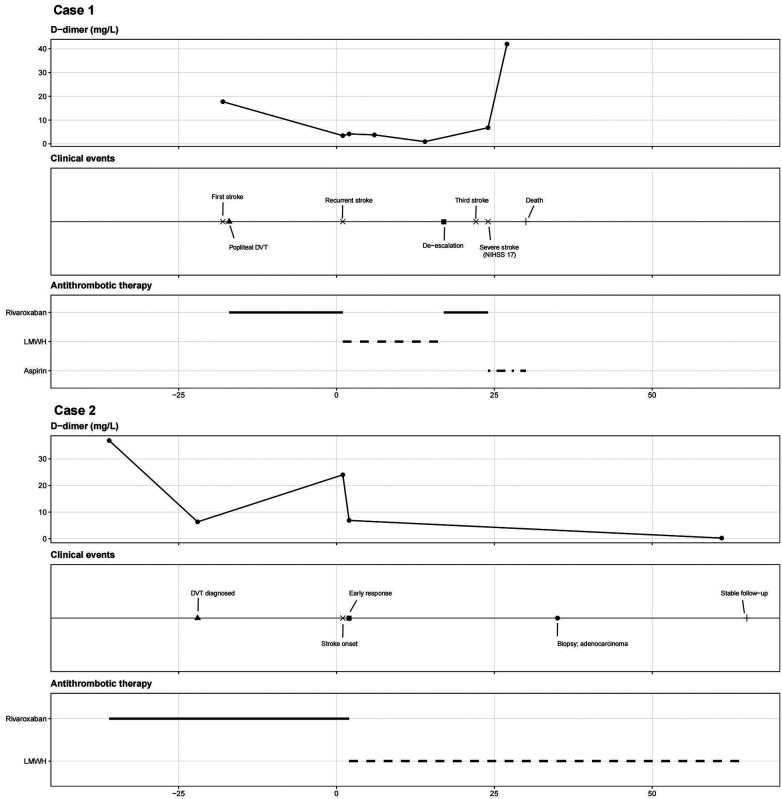
Longitudinal analysis of D-dimer levels, clinical events, and anticoagulation therapy. Case 1 (upper panel) illustrates a phenomenon we term “D-dimer masking,” defined as a transient reduction or normalization of D-dimer levels that does not reflect true resolution of the underlying hypercoagulable state. This pattern suggests a potential discordance between biomarker levels and ongoing prothrombotic activity. All timelines were aligned using the onset of the index stroke as day 0 to improve comparability between cases. Each case is presented across three aligned panels showing (from top to bottom) D-dimer levels, clinical events, and antithrombotic therapy. In contrast, Case 2 (lower panel) shows sustained biomarker suppression and clinical stability following the initiation of LMWH. In the antithrombotic therapy panels, solid horizontal bars represent rivaroxaban/direct oral anticoagulant (DOAC), dashed horizontal bars represent LMWH, and dotted horizontal bars represent antiplatelet therapy. DOAC, direct oral anticoagulant; DVT, deep vein thrombosis; LMWH, low-molecular-weight heparin; NIHSS, National Institutes of Health Stroke Scale.

A comparison of the clinical characteristics, diagnostic findings, treatment strategies, and outcomes of the two cases is provided in Table 1. The temporal relationships among D-dimer dynamics, treatment transitions, and clinical events are illustrated in Figure 2.

**Table 1:** Comparison of the clinical characteristics of the two cases.

Variable	Case 1	Case 2
Age (years)	66	74
Sex	Male	Male
Cancer status	Active intrahepatic cholangiocarcinoma (adenocarcinoma)	Suspected recurrent metastatic gastric adenocarcinoma
Time since initial cancer diagnosis	6 months	26 years
Vascular risk factors	None	Hypertension, diabetes mellitus
Recent venous thromboembolism	DVT (1 month)	DVT (2 weeks)
Prior anticoagulation	Rivaroxaban	Rivaroxaban
Admission NIHSS	6	1
Admission mRS	3	2
Peak D-dimer	41.96 mg/L	36.90 mg/L
Tumor markers	Elevated CEA, CA19-9	Markedly elevated CEA, CA19-9
DWI pattern	Multi-territory infarctions	Multi-territory infarctions
Acute anticoagulation strategy	LMWH (enoxaparin 40 mg (0.4 mL) q12h)	LMWH (enoxaparin 40 mg (0.4 mL) q12h)
Short-term outcome	Recurrent fatal infarction	Clinically stable

DVT, deep vein thrombosis; NIHSS, National Institutes of Health Stroke Scale; mRS, modified Rankin Scale; CEA, carcinoembryonic antigen; CA19-9, carbohydrate antigen 19-9; DWI, diffusion-weighted imaging; LMWH, low-molecular-weight heparin.

## Discussion

This report describes two patients with Trousseau syndrome who developed recurrent multi-territory cerebral infarctions despite ongoing direct oral anticoagulant (DOAC) therapy but exhibited markedly different clinical outcomes following changes in anticoagulation strategies. The integration of serial D-dimer measurements, treatment transitions, and clinical events through a structured timeline analysis highlights the dynamic and unstable nature of malignancy-associated hypercoagulability. Notably, normalization of D-dimer did not reliably indicate sustained control of the hypercoagulable state in Case 1, whereas persistent suppression of D-dimer under low-molecular-weight heparin (LMWH) was associated with clinical stabilization in Case 2. This temporal relationship is further detailed in a structured timeline (Figure 2), which integrates longitudinal biomarker trends with therapeutic adjustments and clinical outcomes. Collectively, these findings suggest a potential dissociation between biomarker normalization and true control of malignancy-associated hypercoagulability.

**Figure 3:**
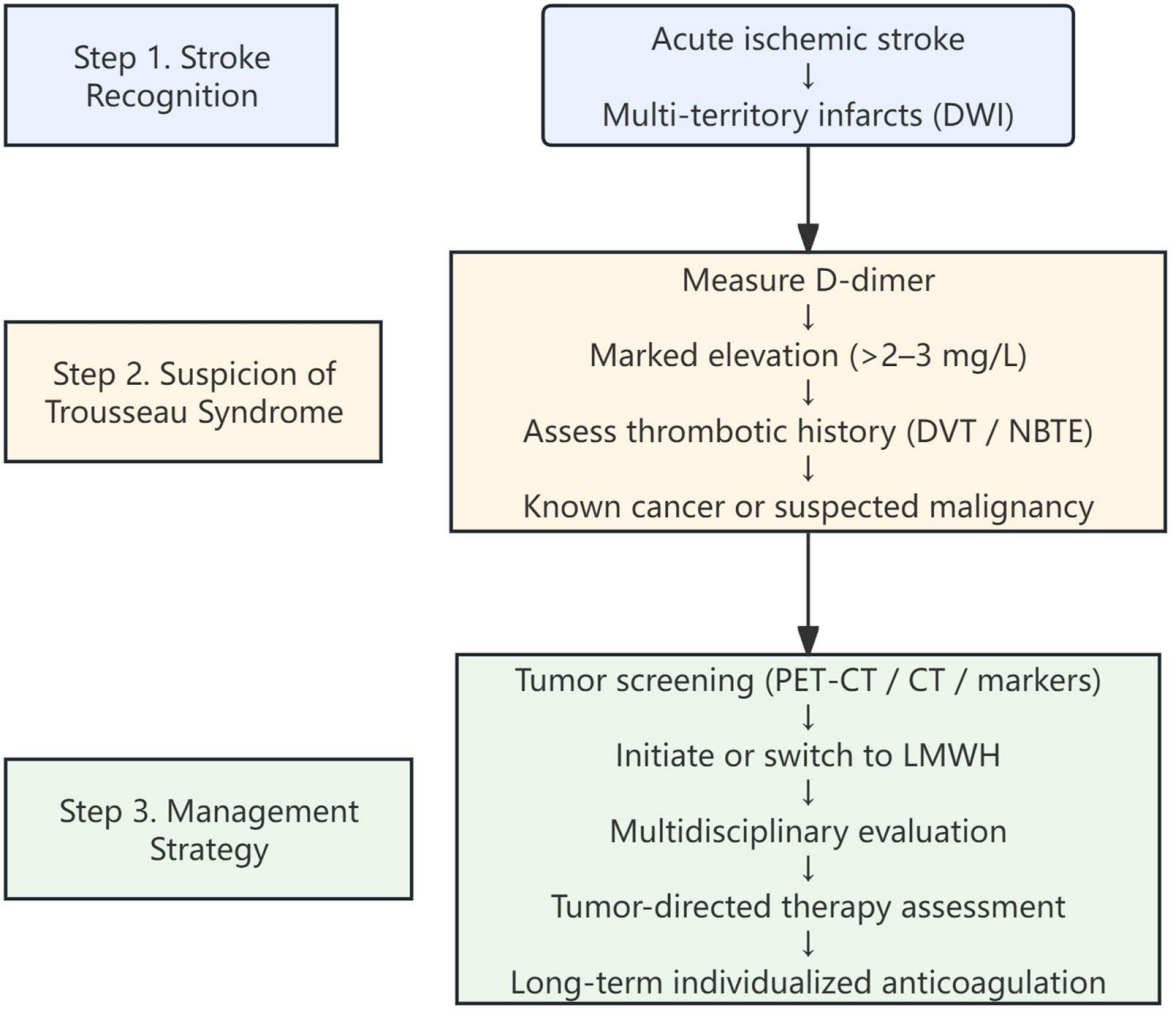
Proposed diagnostic and management workflow for Trousseau syndrome-related stroke. The algorithm integrates key clinical and radiological features, including multi-territory infarctions on diffusion-weighted imaging (DWI), elevated D-dimer levels, thrombotic history, and underlying malignancy status, to guide diagnostic evaluation and therapeutic decision-making. Tumor screening and multidisciplinary assessment are recommended, and low-molecular-weight heparin (LMWH) is emphasized as a key anticoagulation strategy in malignancy-associated hypercoagulability. CT, computed tomography; DVT, deep vein thrombosis; DWI, diffusion-weighted imaging; LMWH, low-molecular-weight heparin; NBTE, nonbacterial thrombotic endocarditis; PET-CT, positron emission tomography-computed tomography.

The pathophysiology of Trousseau syndrome is complex and involves tumor-driven activation of coagulation pathways, including mucin-mediated platelet activation, selectin-dependent interactions, and systemic thrombin generation (2, 3, 5, 6). These mechanisms contribute to widespread microthrombosis and embolic phenomena, often resulting in multi-territory cerebral infarctions (24). In this context, D-dimer serves as a surrogate marker of ongoing coagulation activation and fibrin turnover (20, 21, 23, 25). However, as illustrated in our cases, D-dimer levels may fluctuate dynamically in response to both disease activity and therapeutic interventions, and transient normalization may not necessarily reflect resolution of the underlying prothrombotic state. We propose the concept of “D-dimer masking,” defined as a transient reduction or normalization of D-dimer levels that does not reflect true resolution of the underlying hypercoagulable state. Notably, a potential discordance between markedly elevated tumor markers (e.g., CA19-9) and relatively modest D-dimer levels may reflect differential pathways of thrombogenesis, particularly platelet-rich thrombus formation that is not fully captured by fibrin-based biomarkers. In addition, DOAC therapy may partially suppress fibrin generation without adequately targeting platelet-mediated thrombogenesis, potentially leading to relatively lower D-dimer levels despite ongoing hypercoagulability, thereby creating a misleading impression of disease control. A proposed diagnostic and management workflow for Trousseau syndrome-related stroke, integrating imaging patterns, biomarker assessment, and anticoagulation strategies, is illustrated in Figure 3.

The failure of DOACs in these cases may be attributed to their pharmacological limitations in the setting of malignancy-associated hypercoagulability. While DOACs, such as rivaroxaban, specifically inhibit Factor Xa, they do not address the broader prothrombotic environment characteristic of Trousseau syndrome, such as mucin-mediated platelet activation triggered via P- and L-selectins (5, 6). Given the exceptionally high CA19-9 levels in our patients (>1,200,000 U/mL), we hypothesize that mucin-rich tumor cells directly interact with platelets and neutrophils, effectively bypassing the classical coagulation cascade inhibited by DOACs (2, 6). Conversely, LMWH exerts pleiotropic antithrombotic effects beyond simple factor inhibition, including the blockade of selectin-mediated interactions and the neutralization of tumor-derived microparticles (16, 17). This mechanism may provide superior protection against the widespread microthrombosis driven by tumor-derived mucins, explaining the clinical stabilization observed following the transition to LMWH in our cases (18, 19). Furthermore, recent clinical observations support the concern that DOACs may fail to control the fulminant hypercoagulability encountered in patients with advanced metastatic adenocarcinomas (12, 19).

The optimal anticoagulation strategy for Trousseau syndrome remains controversial. Although DOACs are increasingly used in cancer-associated thrombosis due to their convenience and favorable safety profiles, accumulating evidence suggests that they may be insufficient to fully control malignancy-associated hypercoagulability in certain patients, particularly in the setting of cancer-related stroke (26, 27). In contrast, LMWH has been considered the preferred anticoagulant in this context, not only because of its established efficacy in cancer-associated venous thromboembolism, but also due to its potential to interfere with tumor-mediated prothrombotic pathways, including selectin-dependent interactions (13, 16–18).

Our findings provide clinically relevant insight into this therapeutic dilemma. In Case 1, de-escalation of anticoagulation in the context of apparent biochemical improvement was followed by recurrent stroke and clinical deterioration, whereas in Case 2, sustained anticoagulation with low-molecular-weight heparin (LMWH) was associated with stabilization of both D-dimer levels and clinical status. These contrasting trajectories are consistent with previous observations that malignancy-associated hypercoagulability may not be adequately controlled by DOAC therapy in certain patients (12, 15).

These observations underscore several important clinical implications. First, D-dimer should be interpreted as a dynamic biomarker reflecting ongoing coagulation activity rather than a definitive indicator of resolution, and normalization alone may not reliably justify de-escalation of anticoagulation therapy. Second, LMWH may provide more consistent control of hypercoagulability in selected patients with Trousseau syndrome, particularly in those with recurrent thromboembolic events despite DOAC therapy. Third, anticoagulation strategies should be individualized and guided by the overall clinical course, longitudinal biomarker trends, and underlying disease activity, rather than single time-point measurements. These findings highlight the importance of continuous reassessment of coagulation status in patients with Trousseau syndrome.

This study has several limitations. First, as a case report involving only two patients, the findings are inherently limited by the small sample size and observational nature, precluding causal inference. Second, not all biomarkers of coagulation activation, such as fibrin degradation products, were systematically assessed. Third, nonbacterial thrombotic endocarditis could not be definitively excluded because transesophageal echocardiography was not performed. In addition, medication adherence and potential pharmacokinetic interactions with rivaroxaban were not fully evaluated, which may confound the interpretation of apparent DOAC failure. Furthermore, treatment decisions were influenced by clinical considerations and patient-specific factors, which may limit generalizability. Finally, the follow-up duration in Case 2 was relatively limited, restricting assessment of long-term outcomes. Larger prospective studies are needed to validate these observations and to better define optimal anticoagulation strategies in Trousseau syndrome.

## Conclusion

In conclusion, this report highlights the dynamic nature of malignancy-associated hypercoagulability in Trousseau syndrome and suggests that normalization of D-dimer may not reliably indicate disease control. Sustained anticoagulation with LMWH may offer advantages over DOACs in selected patients with Trousseau syndrome. Careful longitudinal monitoring and individualized treatment strategies are essential to improve clinical outcomes in this high-risk population. These findings support a cautious approach to anticoagulation de-escalation and highlight the importance of sustained and individualized strategies in patients with malignancy-associated stroke. This case report was prepared in accordance with the CARE guidelines.

## Data Availability

The original contributions presented in the study are included in the article, further inquiries can be directed to the corresponding authors.
